# Effect of an entry-to-care intervention on diabetes distress in individuals with newly diagnosed type 2 diabetes: a study protocol for a cluster-randomized trial

**DOI:** 10.1186/s13063-024-07949-6

**Published:** 2024-03-21

**Authors:** Steffan Holst Hansen, Troels Mygind Jensen, Gitte Stentebjerg Petersen, Francois Pouwer, Anders Larrabee Sonderlund, Jens Søndergaard

**Affiliations:** 1 Research Unit of General Practice, University of Southern Denmark, J.B. Winsløws Vej 9A, 5000 Odense, Denmark; 2 Steno Diabetes Center Odense, Odense, Denmark; 3 Department of Psychology, University of Southern Denmark, Odense, Denmark

**Keywords:** Type 2 diabetes, Diabetes distress, Clinical trial, General practice, Complex intervention

## Abstract

**Background:**

Diabetes distress (DD) affects at least 36% of T2DM patients and is often associated with insufficient support and care. This study examines an intervention that targets DD through enhanced cross-sectoral collaboration and treatment during the first 3 months following diagnosis. The intervention aims to improve care and self-management and to reduce DD.

**Methods and intervention:**

The study is designed as a cluster-randomized trial with the intervention focusing on four key elements of diabetes care: effective cross-sectoral communication and information sharing, systematic care, a “one-stop-shop” health screening and start-up conversation at the municipality, and improving patient insights into own care.

This study requires 32 clusters (16/arm) to achieve 80% power and a 5% significance cut-off, with 270 patients required. GP recruitment occurred from May to Dec 2022. Patient recruitment is ongoing from May 2022 to Aug 2023. GPs were randomized 1:1 using computer-generated blocks of six.

Participating GPs are located in Southern Denmark and are not participating in other trials. Patients must be 18 + years of age, have a T2DM diagnosis, and be fluent in spoken and written Danish. DD is the primary outcome and will be measured at baseline, at four months, and again at a 12-month follow-up. Secondary outcomes include quality of care, self-management, quality of life, and clinical factors. Tertiary outcomes comprise depression, stress, resilience, sleep quality, and social network quality.

**Conclusion:**

This study is among the first clinical trials exploring the development of DD from diagnosis to 12 months post-diagnosis. Many previous interventions did not directly target DD as the primary outcome. This research provides new insights into DD progression in patients newly diagnosed with T2DM and examines an intervention designed to lower DD in early diabetes stages, contributing to a better understanding of the development of DD and how this intervention affects patient well-being.

**Trial registration:**

ClinicalTrial.gov NCT05571306. Registered on 07 October 2022.

**Supplementary Information:**

The online version contains supplementary material available at 10.1186/s13063-024-07949-6.

## Strengths and limitations

### Strengths

- Comprehensive intervention: The study employs a multi-faceted approach targeting cross-sectoral communication, systematic care, centralized services, and patient education. This will facilitate a better understanding of how to provide effective holistic care for recently diagnosed T2DM patients.
- Representative sample: The study is based on a large and diverse sample of general practices, municipalities, and hospital units, increasing the generalizability of the findings.
- Multiple outcomes: The study measures a wide range of primary, secondary, and tertiary outcomes, providing a comprehensive understanding of the intervention’s effects on various aspects of patients’ lives.
- Cluster randomization: The use of cluster randomization with stratification for practice size ensures a balanced and homogeneous distribution of intervention and control groups, reducing potential biases.

### Limitations

- The study design does not allow for the blinding of GPs.
- Expected high drop-out rate due to the comprehensive nature of the patient-reported outcomes.
- Exclusion of people who do not speak or write Danish.
- Lack of long-term follow-up beyond 12 months
- Relying primarily on self-reported outcomes may introduce subjectivity and recall bias.

## Introduction

In Denmark, general practitioners (GP) are the primary healthcare providers for most people with type 2 diabetes mellitus (T2DM). However, most patients have continuous contact with different healthcare providers across sectors. This highlights the importance of cross-sectoral collaboration and coordination in the provision of care.

The current Danish clinical guidelines for the treatment of T2DM recommend that patients with new-onset T2DM receive three consultations with their GP to gain information about the disease in general, how it should be managed, and treatment availability. Patients should also be referred to screening for complications [1, 2], health-education programs, and lifestyle interventions offered by the municipality.

The diagnosis of T2DM often confronts patients with a myriad of stressors, ranging from emotional upheavals and disease management challenges to dietary modifications, apprehensions regarding potential complications, and possible alterations in interpersonal relationships [3]. Furthermore, a study conducted by the Steno Diabetes Center Odense (SDCO), in collaboration with the Danish Diabetes Association, sheds light on the immediate post-diagnosis period, revealing a trend of suboptimal quality in consultations and a conspicuous lack of referrals to critical services like health-education programs. Despite their eagerness to engage, patients frequently find themselves excluded from these beneficial programs [4]. This report, while affiliated with our trial and emanating from a survey research methodology, offers valuable insights into the existing gaps in patient support and information provision, highlighting an area ripe for improvement.

This lack of coordination and collaboration in the delivery of diabetes care might restrict early detection and treatment of mental health issues associated with diabetes. This may include depression and/or diabetes distress (DD). DD is a prevalent complication in patients with T2DM, with 36% reporting high levels of distress [5, 6]. DD is characterized by negative emotional experiences resulting from the challenges of living with diabetes, and is associated with low self-care [5, 6], impaired self-management [6], higher HbA1c levels [5], decreased quality of life [6], increased risk of depression [6], and increased all-cause mortality rates among men [7].

Given the negative consequences of DD, early detection and treatment of this condition is imperative. Care should include the following:

- Creating a safe communication environment [8] where patients feel comfortable discussing their concerns, challenges, and needs as they cope with their diagnosis and navigate the health care system.
- Encouraging patient involvement and providing general patient support and health resources (https://www.ncbi.nlm.nih.gov/pmc/articles/PMC5060728/#:~:text=The%20aims%20are%20to%20encourage%2C,280%29%20a%20point, https://www.ncbi.nlm.nih.gov/pmc/articles/PMC6060529/#:~:text=Background,growing%20efforts%20to%20integrate). Focusing on these outcomes can promote a more patient-centered approach to care, which can lead to greater patient motivation, engagement, and empowerment, and thus improve disease management and self-care.

Including these aspects of care to T2DM in the initial months after diagnosis can lead to better self-care and reduced DD [9]. However, there is a lack of interventions that address integrated care pathways for T2DM patients or focus on reducing DD. Further, many existing interventions lack a patient-centered approach, failing to consider patients’ unique needs and situations.

For maximum effect, we argue that DD interventions should be based on improved patient-health professional relationships and communication as well as streamlined cross-sectoral cooperation within the health care system [10]. This project centers on testing an intervention that takes these issues into account.

### Objectives

The objective of this study is to test the effectiveness of a systematic entry-to-care intervention that targets DD. The intervention was developed by Steno Diabetes Centre Odense (SDCO).

#### Aims

- To enhance cross-sectoral collaboration and improve treatment structure during the first 3 months following a T2DM diagnosis.

#### Potential benefits of increased cross-sectoral collaboration

- Improved perceived quality of patient care.
- Enhanced patient self-care and self-management.
- Reduced DD.

#### Potential benefits of decreased DD

- Improved glycemic control.
- Improved diabetes self-care.
- Improved quality of life.
- Reduction in long-term diabetes-related complications.

We acknowledge that patients with better self-care might experience relatively greater improvements in HbA1c levels. As a result, this may decrease DD in these patients as they may feel less stressed and more in control of the disease. In other words, self-care might moderate and/or mediate the intervention effect on DD. Nevertheless, future research may explore these associations.

### Trial design

The trial is designed as a clustered, randomized controlled trial conducted in the primary and secondary sector in the Region of Southern Denmark with a 1:1 allocation ratio.

## Methods: participants, interventions, outcomes

### Study setting

The study is being conducted in the primary and secondary healthcare sectors in the Region of Southern Denmark, which includes 349 general practices with a total of 795 capacities (full-time GPs) and this protocol is written in accordance with the SPIRIT guideline (SPIRIT Guideline). Each capacity makes approximately 6–8 T2DM diagnoses per year, resulting in about 4770 newly diagnosed patients annually. Additionally, the Region of Southern Denmark comprises 22 municipalities and four hospital units, providing a comprehensive healthcare network in the region.

### Eligibility criteria

#### General practitioner eligibility criteria

- General practitioners must practice within the Region of Southern Denmark.
- To prevent potential contamination between current studies, participating GPs must not also be part of the randomized controlled trial study, DICTA [11], which is currently being implemented in the Region of Southern Denmark.

#### Patient eligibility criteria

- Patients must be at least 18 years of age.
- Patients must have been diagnosed with type 2 diabetes by a general practitioner and according to Danish clinical guidelines.
- Patients must have the ability to speak, read, and understand Danish.

### Intervention

The intervention was developed by Steno Diabetes Center Odense with the involvement of both patients and general practitioners. It is designed from the currently existing national guidelines on the treatment of T2DM (https://www.dsam.dk/vejledninger/type2/saerlige-udfordringer-hos-sarbare-patienter), to support people with T2DM in the first 3 months following their diagnosis. This comprehensive approach includes four fundamental elements.

1. Improving cross-sectoral communication and information sharing

To improve cross-sectoral collaboration, SDCO has organized a series of face-to-face meetings involving general practitioners, municipal representatives, and representatives from regional hospitals. These gatherings aim to foster interpersonal relationships, promote knowledge sharing, and facilitate discussions regarding the unique healthcare services that each sector offers to people with newly diagnosed T2DM.

2. Systematic care

In Denmark, comprehensive clinical guidelines for the treatment of T2DM are currently available. While these guidelines provide valuable information, their extensive nature and complexity represents a barrier to implementation. To address this issue, and to ensure systematic and uniform care for people recently diagnosed with T2DM, SDCO has developed Quick Guides, which offers concise and easily understandable resources specifically designed for general practitioners. This efficient tool aims to streamline the application of best practices in T2DM management. The guidelines establish a framework outlining the essential information that patients should receive within the initial 3 months following their diagnosis. Moreover, to optimize the implementation process, SDCO plans to conduct individual meetings with each general practitioner, allowing for a tailored approach that caters to their specific needs.

3. A “one-stop-shop” and a start-up conversation at the municipality

Each patient will be referred for an introductory consultation at their respective municipality to evaluate the relevance and necessity of health-related programs tailored to their individual needs. Furthermore, all patients will be directed to a “one-stop-shop” health-screening appointment, where they can undergo multiple essential examinations, such as blood sample collection, blood pressure measurement, and eye and foot screenings. These referrals aim to promote consistency in T2DM treatment and establish a foundation for enhanced and optimal care.

Unlike the current standard care where patients are required to independently initiate contact for blood sample tests, arrange screenings for potential complications such as eye and foot issues, and often face lengthy waiting times for these services, the “one-stop-shop” approach aims to consolidate these essential services into a single, comprehensive appointment.

4. Improving patient overview

In partnership with individuals affected by type 2 diabetes mellitus, SDCO has created patient information materials, which have been designed to provide patients with a structured, coherent, and comprehensive overview during the initial stages post-diagnosis. The materials include a checklist and essential information about the disease and were developed to assuage the uncertainty that patients may encounter after being diagnosed. These materials are thus designed to enhance patients’ understanding of the nature, symptomatology, and treatment of T2DM. As a result, this initiative is expected to alleviate diabetes-related distress by bolstering patients’ sense of confidence and knowledge about their chronic disease.

### Outcomes

#### Primary outcome

This study primarily investigates longitudinal changes in DD at baseline, 4 months, and 12 months. The DDS is a validated and reliable 17-item self-report tool that measures emotional burden, regimen-related distress, physician-related distress, and interpersonal distress [5, 10, 12]. Participants rate items on a six-point Likert scale, with overall and subscale scores calculated by averaging responses. Physician-related distress might be a key focus in the analysis, as it may be directly influenced by the intervention.

#### Secondary outcomes (measured at baseline, 4, and 12 months)

Several secondary outcomes will be assessed, including the perceived quality of care using study-specific items, patients’ ability to self-manage using the Patient Activation Measure (PAM) [13], quality of life using the 12-item Short Form (SF-12) [14], patients’ self-care ability using the Summary of Diabetes Self-Care Activities Measurement (SDSCA) [15], and clinical components such as HbA1c, blood pressure, and blood lipids will be measured.

#### Tertiary outcomes (measured at baseline, 4, and 12 months)

Depression, stress, and resilience will be assessed using the Major Depression Inventory (MDI) [9], the two single-item measures of psychosocial stress [16], and the Connor-Davidson Resilience Scale (CD-RISC-10) [17]. Furthermore, the extent and quality of social networks will be assessed using the Lubben Social Network Scale (Lubben-6) [18] and sleep quality and quantity will be measured using the Pittsburgh Sleep Quality Index (PSQI).

For a complete overview of the primary, secondary, and tertiary outcomes, see Table 1.

**Table 1:** Outcome measures

Outcome measure	Description
Diabetes Distress Scale (DDS)	Diabetes Distress Scale is a 17-item questionnaire that assesses distress associated with diabetes. It is divided into four subscales: emotional burden, physician-related distress, regimen-related distress, and interpersonal distress. The items are scored on a 6-point Likert scale ranging from 1 (no problem) to 6 (serious problem). Higher scores indicate greater distress. It has good internal consistency with a Cronbach’s alpha of 0.88–0.93 [10].
Patient Activation Measure (PAM)	The PAM is a 13-item questionnaire that measures patients’ knowledge, skill, and confidence in managing their health. Items are scored on a 4-point Likert scale ranging from 1 (strongly disagree) to 4 (strongly agree). The total score is converted to an activation score ranging from 0 to 100, with higher scores indicating greater activation. The PAM has a Cronbach’s alpha of 0.87 [13].
Short Form (SF-12)	The SF-12 is a 12-item generic health-related quality of life measure, derived from the longer SF-36 questionnaire. It consists of two summary scores: the Physical Component Summary (PCS) and the Mental Component Summary (MCS). Scores range from 0 to 100, with higher scores indicating better health. The SF-12 has demonstrated good internal consistency with a Cronbach’s alpha of 0.81 for PCS and 0.76 for MCS [19].
Summary of Diabetes Self-Care Activities Measure (SDSCA)	The MDI is a 12-item self-report questionnaire that measures symptoms of major depression. The questionnaire covers the ten ICD-10 and DSM-IV diagnostic criteria for depression. This MDI is scored on a 6-point Likert scale ranging from 0 (not at all) to 5 (all the time). Total scores range from 0 to 50, with higher scores indicating greater depressive symptoms. The MDI has demonstrated good internal consistency with a Cronbach’s alpha of 0.89 [15].
Major Depression Inventory (MDI)	The MDI is a 12-item self-report questionnaire that measures symptoms of major depression. The questionnaire covers the ten ICD-10 and DSM-IV diagnostic criteria for depression. Items are scored on a 6-point Likert scale ranging from 0 (not at all) to 5 (all the time). Total scores range from 0 to 50, with higher scores indicating greater depressive symptoms. The MDI has demonstrated good internal consistency with a Cronbach’s alpha of 0.89 [20].
2 single-item measures of psychosocial stress	These two single-item measures assess perceived stress and coping resources. The first item measures the level of perceived stress on a scale from 1 (not stressed) to 10 (extremely stressed). The second item measures the perceived ability to cope with stress on a scale from 1 (not able to cope) to 10 (very able to cope). Although single-item measures may have lower reliability than multi-item measures, it has been used in several research settings to assess psychosocial stress [16].
Connor-Davidson Resilience Scale (CD-RISC-10)	The CD-RISC-10 is a shorter, 10-item version of the original 25-item Connor-Davidson Resilience Scale that measures resilience, or the ability to cope with adversity. Items are scored on a 5-point Likert scale ranging from 0 (not true at all) to 4 (true nearly all the time). Total scores range from 0 to 40, with higher scores indicating greater resilience. The CD-RISC-10 has demonstrated good internal consistency with a Cronbach’s alpha of 0.85 [17, 21]
Lubben Social Network Scale-6 (LSNS-6)	The LSNS-6 is a shorter, 6-item version of the original 10-item Lubben Social Network Scale that assesses social network size and social support. It consists of two subscales: family and friends. Items are scored on a 6- point scale ranging from 0 (none) to 5 (nine or more). Total scores range from 0 to 30, with higher scores indicating larger social networks and greater support. The LSNS-6 has good internal consistency with a Cronbach’s alpha of 0.80 for the family subscale and 0.83 for the friend’s subscale [18].
Pittsburgh Sleep Quality Index (PSQI)	The PSQI is a 19-item self-report questionnaire that assesses sleep quality and disturbances over the past month. It consists of seven component scores: subjective sleep quality, sleep latency, sleep duration, habitual sleep efficiency, sleep disturbances, use of sleeping medication, and daytime dysfunction. Component scores range from 0 to 3, and the total score ranges from 0 to 21, with higher scores indicating poorer sleep quality. A total score greater than five suggests significant sleep disturbances. The PSQI has demonstrated good internal consistency with a Cronbach’s alpha of 0.83 [22].

#### Items created for the study (measured at baseline, 4, and 12 months)

These items were developed to address experiences of care that the aforementioned questionnaires do not cover. Specifically, items were developed based on other Danish patient surveys and validated [4, 23] in terms of respondent interpretation and understanding of each item.

### Sample size

The required minimum number of clusters (a cluster being one general practice, which can include several general practitioners) was calculated, based on the means and standard deviations (changes in DD from baseline to follow-up) reported in the REDEEM trial conducted by Fisher et al. [24]. Assuming a low intraclass correlation of 0.05 (https://www.ncbi.nlm.nih.gov/pmc/articles/PMC4983799/) and a mean group cluster size of 6 T2DM patients observed per year in the control and intervention groups, it was determined that 32 clusters (16 in each arm) are needed to achieve power at 80% and a statistical significance level of 5%. Although a dropout rate of 40% is considered high, it is likely for the present study due to the comprehensive patient-reported outcomes, which require three follow-ups. As such, a total of 270 patients are required for analysis.

### Recruitment

#### General Practitioners

Strategies to recruit GPs to the project were: telephone calls, followed up with emails and postal mail. To maintain engagement, GPs within the control group will be offered the intervention training and resources after the trial is complete. GP recruitment took place from May to December 2022.

### Patients

Participating GPs began recruiting patients in May 2022 and ended late August 2023. Participants can access the patient-reported outcome questionnaire via e-Boks, a widely used Danish digital platform for secure communication with public authorities and private companies.

Patients lacking access to e-Boks or who were unable to complete the electronic questionnaire, will be provided a hard copy by their GP or schedule a phone interview with a member of the research team.

### Participant timeline

**Fig. 1:**
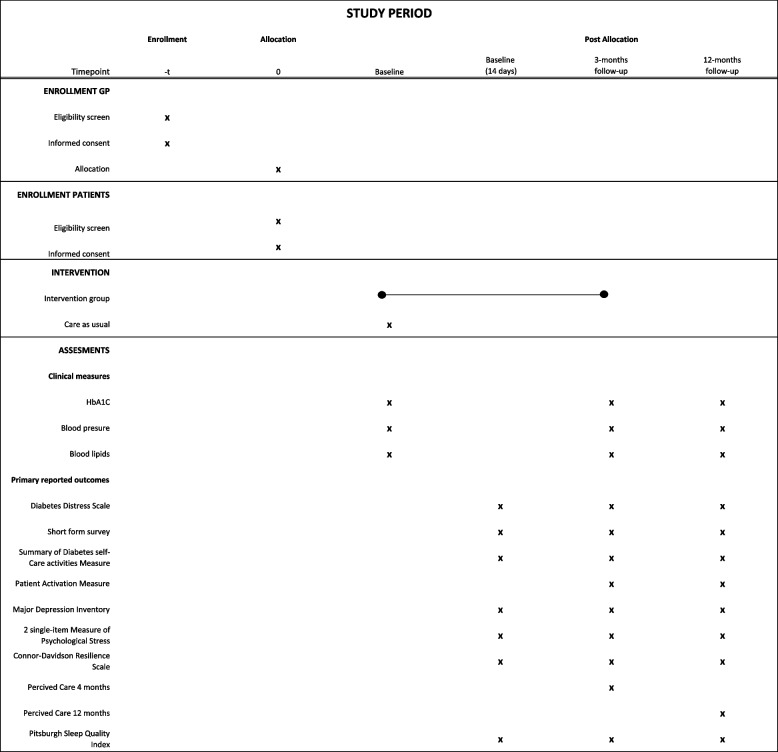
Time schedule of enrolment

The time schedule for the enrolment of both GPs and patients is presented in Fig. 1.

## Methods: assignment of interventions (for controlled trials)

### Allocation

GPs were randomized into intervention or control clusters at a 1:1 allocation ratio using a computer-generated block size of six. The GPs were stratified into two groups, taking their capacities into account (One with general practitioners with 1 or 2 capacities, and another with 3 or more capacities). This approach aimed to ensure a homogeneous distribution of small and large general practices in each intervention arm. A statistician blinded to the practice identities performed the allocation, and the process was concealed within sealed envelopes. These sealed envelopes where then opened by SDCO in the correct order, when allocating GPs, to minimize the risk of unintentional allocation bias or interference.

The control group in this study is subject to “usual care,” which encompasses the routine practices and treatments typically administered by GPs or a diabetic nurse for patients diagnosed with T2DM.

Usual care, by its nature, is subject to variation as it is contingent upon the individual practices of the GPs, their interpretation of the patient’s needs, and adherence to national guidelines. In Denmark, there are established clinical guidelines for the management and treatment of T2DM, and while we anticipate that several GPs in the control group will adhere to these guidelines, it is important to acknowledge that the extent and nature of adherence may vary.

In the context of these national guidelines, usual care may involve the patient receiving three consultations to gather information about T2DM, understand how to manage the disease, learn about available treatment options, and receive referrals for screenings of potential complications, health-education programs, and lifestyle interventions. However, due to the variance in practices across different healthcare providers, the application of these guidelines may not be uniform.

### Blinding

The study design does not allow blinding of GP. However, patients are blinded as they enter the study unaware of their treatment, ensuring genuine responses. Data analysts are also kept in the dark, maintaining unbiased data interpretation.

## Methods: data collection, management, and analysis

### Data collection methods

The present study utilizes an electronic patient-reported questionnaire as the primary method for data collection, with the option of completing a hard copy or telephone version of the questionnaire. The questionnaire was designed to gather information on outcomes that are deemed essential to the study’s objectives. These outcomes were selected based on their validation as reliable tools for measuring key outcomes. To ensure participant confidentiality and data integrity, each participant is assigned a unique, anonymized ID, concealing identities from data analysts.

### Data management

The present study utilizes e-Boks and the Open Patient data Explorative Network (OPEN) for the collection of data. The survey instrument has been developed using OPEN REDCap, and the resulting data is stored on the server provided by OPEN. For security purposes, the data will be extracted from the OPEN server and transferred to a secure S4 server provided by SDU. All data management and handling will be carried out exclusively on the S4 server, adhering to the most stringent standards of data security and confidentiality.

### Statistical methods

To evaluate the effectiveness of the intervention, intention-to-treat analysis will be performed. The Generalized Estimating Equations model (GEE), which accounts for cluster randomization, will be utilized for data analysis. The model will be adjusted for the main potential confounding variables. Data analysis for this research will be conducted using RStudio (RStudio Team, 2022), a widely used open-source integrated development environment for the R programming language. Statistical significance set at *P* < 0.05. Ad-hoc analyses will also be conducted as necessary to support future hypotheses. Missing data, will adhere to the guidelines specific to each patient-reported outcome measure, ensuring appropriate and validated methods are utilized for each case. This approach ensures the integrity and validity of our data, even when some values are missing.

Descriptive statistics, we provide measures of central tendency (mean or median) and dispersion (standard deviation or interquartile range) for continuous variables, and frequencies and percentages for categorical variables. This comprehensive presentation of data will facilitate a clear and thorough understanding of the demographic and clinical characteristics of the study population.

## Discussion

The primary aim of this study is to evaluate a cross-sectoral intervention targeting DD in newly diagnosed T2DM patients through increased cross-sectoral collaboration. The anticipated results of the study will comprise effective tools for improving overall collaboration between health care sectors. These tools are envisioned for direct incorporation into routine clinical practice and will thus contribute to improvements in the overall health and well-being of patients.

Despite every effort to minimize bias, this study has some limitations. The lack of blinding of GPs in the study not only introduces the possibility of differential intervention delivery but also raises concerns about selection bias in the inclusion of patients. General practitioners might inadvertently select patients with certain characteristics or conditions that could influence the study outcomes. Despite the potential for selection bias due to the lack of blinding of GPs, the study has communicated the importance of including all patients with clear and precise instructions. Emphasizing the significance of a diverse and representative patient population is crucial for the study’s success, as it helps to mitigate the risks associated with selection bias and ensures the findings are more generalizable. Another limitation is that the study includes only patients who are fluent in Danish and thus presumably disproportionately excludes people from immigrant and ethnic minority groups. However, research from the USA indicates that minoritized ethnic groups are often at increased risk of diabetes distress [25]. An English questionnaire translation was considered, but as ethnic minorities in Denmark primarily speak variations of either Hindi or Arabic, a multilingual translation was required to effectively deal with this limitation. This, however, was beyond our funding capacity and thus not possible Lastly, using PROs as the primary measure presents limitations, including subjectivity influenced by personal biases, emotions, and beliefs, which can result in inconsistent reporting. Recall bias may affect accuracy and reliability, as patients might struggle to remember details accurately, leading to underreporting or overreporting of symptoms. However, as DD only can be measured using PROs, they are essential and offer unique insights into patients’ lived experiences which cannot be measured from clinical outcomes.

## Conclusion

To our knowledge, this study is one of the first clinical trials investigating the development of DD from the time of diagnosis to 12 months post-diagnosis. This study offers valuable new insights into the progression of DD in patients recently diagnosed with type 2 diabetes and examines the effects of an intervention specifically designed to lower DD during the early stages of diabetes through a complex intervention, thereby contributing to a deeper understanding of how such interventions can impact patients’ well-being.

## Trail status

This is Protocol version 1.1, as of October 24, 2023. The process of recruiting GPs for the study kicked off in May 2022 and ended in December 2022. In tandem with GP enlistment, we started recruiting patients in May 2022. The recruitment of patients ended in late August 2023.

The timing of this protocol submission comes a bit later in the process, after we completed GP recruitment but before our final patient visit. This is largely due to the fact that this study is a key part of a Ph.D. journey, which meant that academic commitments and scheduling made it challenging to submit the protocol earlier. Rest assured, this timing has had no impact on the diligence of our research, the quality of the study, or our steadfast commitment to uphold ethical standards throughout the process.

### Supplementary Information

**Additional file 1.**

## Data Availability

The final trial dataset will be available for researchers and statisticians at the Research Unit of General Practice at the University of Southern Denmark.
