# Correction: Effect of an entry-to-care intervention on diabetes distress in individuals with newly diagnosed type 2 diabetes: a study protocol for a cluster-randomized trial

**DOI:** 10.1186/s13063-024-08075-z

**Published:** 2024-04-04

**Authors:** Steffan Holst Hansen, Troels Mygind Jensen, Gitte Stentebjerg Petersen, Francois Pouwer, Anders Larrabee Sonderlund, Jens Søndergaard

**Affiliations:** 1 Research Unit of General Practice, University of Southern Denmark, J.B.Winsløws Vej 9A, 5000 Odense, Denmark; 2 Steno Diabetes Center Odense, Odense, Denmark; 3 Department of Psychology, University of Southern Denmark, Odense, Denmark

**Correction** **:** **Trials 25, 207 (2024)**

**https://doi.org/10.1186/s13063-024-07949-6**

Following publication of the original article [1], we have been notified that the name of the 5th author was incorrectly spelled. Additionally, his part of his last name was tagged as first name.

Originally spelled name: Anders L. Sønderlund

Correct spelling of the name: Anders Larrabee Sonderlund

The original article has been corrected.
